# Adipose tissue macrophages as potential targets for obesity and metabolic diseases

**DOI:** 10.3389/fimmu.2023.1153915

**Published:** 2023-04-19

**Authors:** Xirong Li, Yakun Ren, Kewei Chang, Wenlong Wu, Helen R. Griffiths, Shemin Lu, Dan Gao

**Affiliations:** ^1^ Institute of Molecular and Translational Medicine, School of Basic Medical Sciences, Xi’an Jiaotong University Health Science Center, Xi’an, China; ^2^ Key Laboratory of Environment and Genes Related to Diseases (Xi’an Jiaotong University), Ministry of Education, Xi’an, China; ^3^ Department of Human Anatomy, Histology and Embryology, School of Basic Medical Sciences, Xi’an Jiaotong University Health Center, Xi’an, China; ^4^ Swansea University Medical School, Swansea University, Swansea, United Kingdom; ^5^ Department of Biochemistry and Molecular Biology, School of Basic Medical Sciences, Xi’an Jiaotong University Health Science Center, Xi’an, China

**Keywords:** macrophages, adipose tissue, plasticity, obesity, metabolic diseases

## Abstract

Macrophage infiltration into adipose tissue is a key pathological factor inducing adipose tissue dysfunction and contributing to obesity-induced inflammation and metabolic disorders. In this review, we aim to present the most recent research on macrophage heterogeneity in adipose tissue, with a focus on the molecular targets applied to macrophages as potential therapeutics for metabolic diseases. We begin by discussing the recruitment of macrophages and their roles in adipose tissue. While resident adipose tissue macrophages display an anti-inflammatory phenotype and promote the development of metabolically favorable beige adipose tissue, an increase in pro-inflammatory macrophages in adipose tissue has negative effects on adipose tissue function, including inhibition of adipogenesis, promotion of inflammation, insulin resistance, and fibrosis. Then, we presented the identities of the newly discovered adipose tissue macrophage subtypes (e.g. metabolically activated macrophages, CD9^+^ macrophages, lipid-associated macrophages, DARC^+^ macrophages, and MFe^hi^ macrophages), the majority of which are located in crown-like structures within adipose tissue during obesity. Finally, we discussed macrophage-targeting strategies to ameliorate obesity-related inflammation and metabolic abnormalities, with a focus on transcriptional factors such as PPARγ, KLF4, NFATc3, and HoxA5, which promote macrophage anti-inflammatory M2 polarization, as well as TLR4/NF-κB-mediated inflammatory pathways that activate pro-inflammatory M1 macrophages. In addition, a number of intracellular metabolic pathways closely associated with glucose metabolism, oxidative stress, nutrient sensing, and circadian clock regulation were examined. Understanding the complexities of macrophage plasticity and functionality may open up new avenues for the development of macrophage-based treatments for obesity and other metabolic diseases.

## 1 Introduction

Obesity has become a global pandemic, and its prevalence is increasing at an alarming rate (1). The rise in the prevalence of obesity significantly increases the risk of chronic metabolic diseases, such as cardiovascular disease, diabetes, hypertension, and cancer, and have a detrimental impact on both health and quality of life. Clarifying the pathogenesis of obesity is crucial for the prevention, treatment, and management of chronic metabolic diseases associated with obesity.

Obesity is characterized by an increase in the accumulation of macrophages in adipose tissue, which is accompanied by adipose tissue dysfunction, such as reduced adipogenesis and lipid storage capacity, adipocyte necrosis, inflammation, insulin resistance, and fibrosis (2). Adipose tissue stores excess energy in two ways: adipocyte hypertrophy and proliferation. Adipocyte proliferation is the healthy development of adipose tissue driven by preadipocyte proliferation and differentiation, whereas adipocyte hypertrophy is a pathological expansion of existing adipocytes with increased lipid storage and is closely related to adipocyte dysfunction (3). Hypertrophic adipocytes secrete a large number of chemokines, recruit immune cells, particularly macrophages, and cause chronic low-grade inflammation, insulin resistance, and the release of a large amount of free fatty acids into the circulation, eventually leading to obesity-related metabolic disorders (4).

A growing body of studies have indicated that innate immune cells play an important role in modulating adipose tissue activities during obesity (5). Among these cells, macrophages were the first and most important immune cells discovered infiltrating adipose tissue during obesity (6, 7). Macrophage infiltration has a significant impact on adipose tissue function and is a major cause of obesity-related metabolic diseases. Therefore, understanding the molecular mechanisms governing adipose tissue macrophages is critical for the prevention and treatment of obesity and other related metabolic diseases. Here, we review the current literature on adipose tissue macrophages with a particular emphasis on the heterogeneity and polarization of these cells during obesity in adipose tissue. We discuss the fundamental roles of macrophages in adipose tissue, highlighting macrophage-targeting strategies and assessing their therapeutic potential for treating obesity and related metabolic diseases.

## 2 Adipose tissue macrophages

### 2.1 Increased macrophage recruitment to adipose tissue in obesity

The primary sources of adipose tissue macrophages are tissue-resident macrophages and monocyte-derived recruited macrophages. Unlike most tissue-resident macrophages, which are derived from yolk sac primitive precursors and function to regulate tissue remodeling and maintain tissue homeostasis (8), a recent fate mapping study revealed that adipose tissue resident macrophages are derived from definitive embryonic hematopoietic precursors (9). These resident ATMs are phenotypically F4/80^hi^CD11b^+^CD169^+^ cells that can be further subdivided into three subtypes: MHCII^low^, MHCII^+^CD11c-, and MHCII^+^CD11c^+^. In response to HFD, the MHCII^+^CD11c^+^ ATMs were rapidly increased in adipose tissue and replenished by bone marrow-derived monocytes, implying that recruited monocytes are the major cells contributing to increased ATMs in obesity.

Infiltration of monocyte-derived macrophages into adipose tissue during obesity was firstly reported in mouse models obesity and humans in 2003 (6, 7). The infiltrated macrophages were derived from bone marrow (7) and were contributed by increased diapedesis of blood monocytes (10). In contrast, weight loss by surgery reduced macrophage infiltration in adipose tissue of patients with obesity (11). Chemokine and its receptor interaction play crucial roles in the recruitment of circulating monocytes into adipose tissue during obesity. For example, monocyte chemoattractant protein (MCP-1 or CCL2), a chemokine produced in both adipocytes and the stromal vascular (SV) portion of adipose tissue, is significantly elevated in both blood and adipose tissue in obesity (12–17). Mice lacking CCL2 (18) or its receptor, CC chemokine receptor 2 (CCR2) (19) or using CCR2 inhibitor (20, 21), have lower adipose tissue macrophage infiltration and improved metabolic function in *db/db* and HFD-induced obese mice. Conversely, mice overexpressing CCL2 in adipose tissue have enhanced macrophage infiltration into adipose tissue and an unfavorable metabolic profile (18, 22). Moreover, mice with CCR2 deficiency in bone marrow cells or macrophages had lower macrophage numbers in adipose tissue after high-fat diet (HFD) feeding, indicating that CCR2 plays a crucial role in macrophage recruitment into adipose tissue during obesity (23, 24).

In addition to CCL2/CCR2, other chemokines and their receptors may play a role in the increased macrophage accumulation in adipose tissue in obesity. For instance, CCL chemokines (such as CCL3, CCL4, CCL5, CCL7, CCL8, CCL11, CCL18) and its receptors (such as CCR1, CCR3 and CCR5) have been linked to increased adipose tissue in obese (25) and human individuals (26, 27). Indeed, a dual CCR2/5 antagonist significantly reduces M1 macrophage infiltration into adipose tissue in HFD-induced obese mice, as well as improving adipose tissue inflammation and insulin resistance (IR) (28). Furthermore, CXCL12 produced by adipocytes interacts with its receptor CXCR4 to mediate macrophage recruitment into adipose tissue during HFD-induced obesity (29). In addition, other chemokines such as haptoglobin and C3a have also been reported to mediate macrophage recruitment into adipose tissue during obesity (30, 31). These studies taken together have demonstrated the therapeutic potential of focusing on macrophage recruitment into adipose tissue.

### 2.2 Adipose tissue macrophages polarized to pro-inflammatory phenotype in obesity

Increased macrophage infiltration into adipose tissue forms a crown-like structure (CLS) around necrotic adipocytes (32, 33). The number of CLS is strongly correlated with the expression of inflammatory cytokines like TNF-α (32), indicating that infiltrating macrophages have a pro-inflammatory effect on adipose tissue in obesity. Lumeng et al. used PKH26 dye to label resident macrophages in adipose tissue and found that newly recruited adipose tissue macrophages (ATMs) in HFD-induced obese mice had a pro-inflammatory M1 phenotype (F4/80^+^CD11c^+^), whereas resident macrophages had an alternative activated M2 phenotype (F4/80^+^CD206^+^) (34–36).

Further examination of CD11c^+^ ATMs from epididymal WAT (eWAT) revealed a mixed M1/M2 profile that was divided into three subtypes: resident ATMs as MGL1^+^CD11c^-^ expressing cells, CLS-associated MGL1^-^/CD11c^+^ ATMs, and MGL1^med^/CD11c^+^ ATMs (37). Similar to this work, resident ATMs in human adipose tissue have been shown to display M2 markers like CD206 and CD163, but they are also able to produce inflammatory cytokines (38, 39), indicating that these ATMs are mixed M1- and M2-polarized. Additionally, the number of ATMs in subcutaneous and omental adipose tissue of patients with obesity is higher than in lean subjects (40, 41). These findings collectively indicate that adipose tissue remodeling in obesity is connected to both an M1 and M2 progression.

Moreover, macrophage infiltration into adipose tissue during obesity is preferentially located in visceral adipose tissue in humans (42–44) and mice (33, 45), implying that visceral adipose tissue is the major adipose depot harboring the pro-inflammatory macrophages in obesity. The pro-inflammatory ATMs are one of the key cell types responsible to produce pro-inflammatory cytokines such as TNF-α, IL-1β, and IL-6, which contribute to obesity-related adipose tissue inflammation. In addition to the recruitment of circulating monocytes into adipose tissue, a local proliferation of macrophages in CLS also contributes to the increased ATMs in adipose tissue during obesity (46–48). These proliferating macrophages express M2 macrophage markers including CD206 and CD301 and form resident ATMs in the interstitial space (49). Even though these proliferating macrophages are M2 phenotype, their presence maintained adipose tissue inflammation in obese mice even after weight loss (50).

The accumulation of macrophages in adipose tissue is not only a defining feature of obesity, but also a major cause of obesity-related metabolic diseases such as liver steatosis and IR (51–57). As a result, reducing the number of macrophages in adipose tissue slows the onset of obesity and improves insulin sensitivity and glucose metabolism (58, 59), indicating that macrophages in adipose tissue play crucial roles in the development of obesity and metabolic disorders.

## 3 Adipose tissue macrophage subtypes and functions in adipose tissue

### 3.1 Newly identified macrophage subtypes in adipose tissue

In addition to previously classified pro-inflammatory and alternatively activated macrophages using F4/80 and CD11c or CD206 markers, a new class of ATMs known as M3 ATMs (CD11c^-^CD206^-^MGL1^-^) that also localize to the CLS and uniquely express chemokine receptor Ccr7 has been reported (60). The presence of M3-like ATM suggests that different pathways may contribute to macrophage inflammation in the context of obesity. Additionally, another new type of ATM known as metabolically activated macrophages (MMe) were reported, which is produced when exposed to high levels of glucose, insulin, and palmitate. Rather than expressing classical M1 markers, MMe overexpress ATP binding cassette transporter (ABCA1), cluster of differentiation 36 (CD36), and perilipin 2 (PLIN2), which are regulated by peroxisome proliferator activated receptor gamma (PPARγ) (61). Moreover, MMe macrophages accumulated in CLS showed both beneficial and detrimental effects in response to high-fed diet feeding (62). For example, during the early stages of HFD-induced obesity, MMe macrophages increased adipose tissue inflammation by upregulating inflammatory markers such as TNF-α, IL-6, and IL-1β, as well as genes involved in lipid metabolism. In contrast, despite strong expression of pro-inflammatory and lipid metabolism genes in MMe macrophages, they are more active in the clearance of dead adipocytes *via* lysosomal exocytosis, hence inhibiting ectopic fat accumulation and IR in late-onset HFD-induced obesity. Mechanistically, TLR2, NOX2 and MyD88 have been proposed to modulate the positive and negative impact of MMe macrophages in HFD-induced obesity. Subsequent research suggested that MMe aggregation in breast adipose tissue may play a role in the development of triple-negative breast cancer (63).

**Figure 1:**
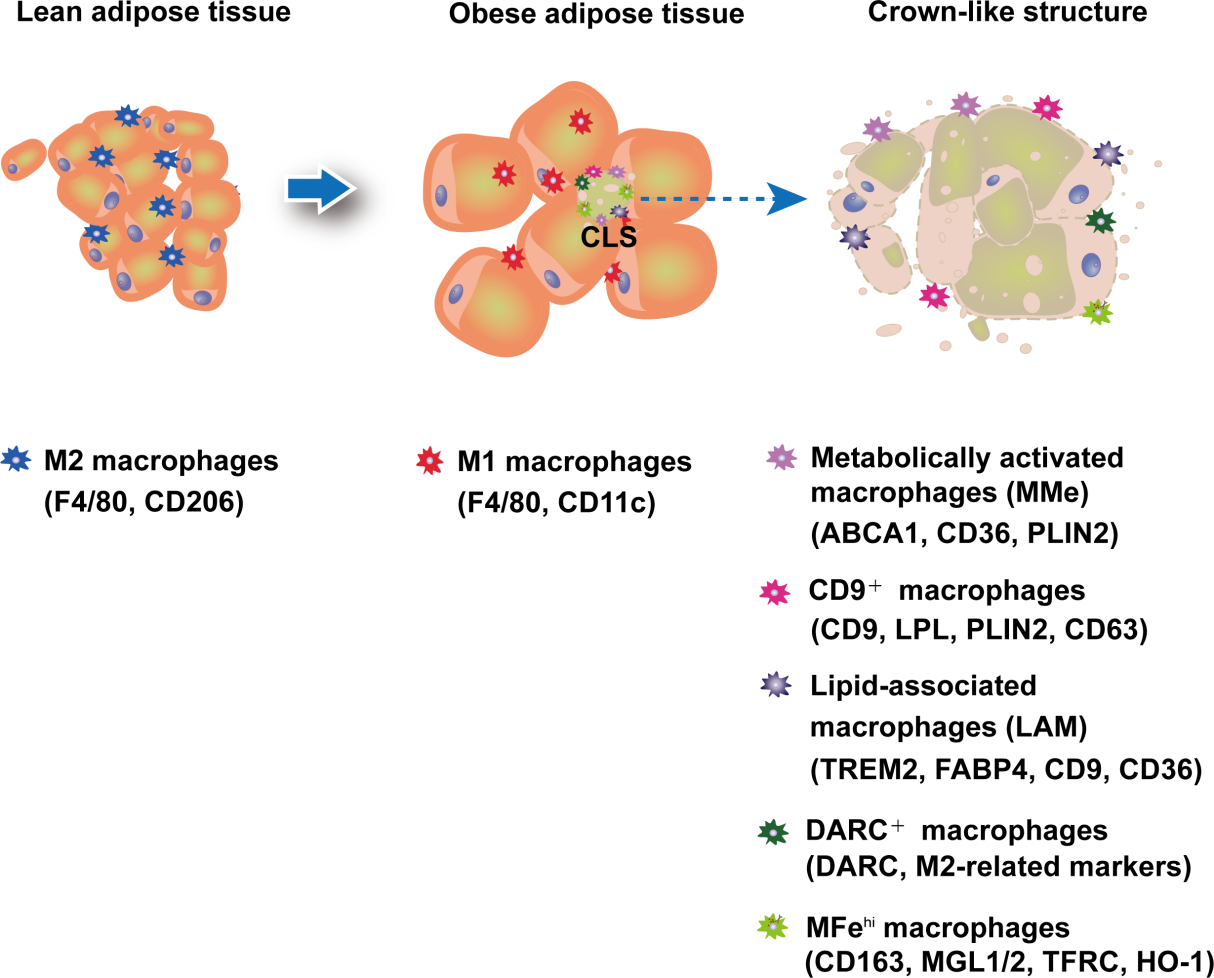
ATMs plasticity in adipose tissue. In lean adipose tissue, anti-inflammatory M2-like macrophages are predominant and maintain homeostasis. In obese adipose tissue, an increase in pro-inflammatory M1 ATMs forms a crown-like structure (CLS) surrounding dead adipocytes. Recent research has uncovered new macrophage subtypes, particularly in CLS.

Recent research using single-cell sequencing has revealed a much broader range of ATM phenotypes (**Figure 1**; **Table 1**). For example, CD9^+^ATM, which also localizes in CLS in both mice and humans, was discovered to contain large amounts of intracellular lipids in lysosomal-like structures and to express genes associated with lysosome-dependent lipid metabolism, may have the same capacity as MMe to clear dead adipocytes *via* the lysosomal pathway. However, CD9^+^ATM is distinct from MMe because it contains traditional M1/M2 markers like CD206 and CD11b (64). Adoptive transfer of CD9^+^ ATM to lean mice leads to the up-regulation of genes related with obesity, suggesting that CD9^+^ ATM may promote the development obesity and metabolic diseases (64). Triggering receptor expressed on myeloid cells 2 (TREM2), a pathologically induced immune signaling in Alzheimer’s disease, metabolic diseases, and cancer, has been found to express in ATMs (69). A new subtype of macrophages termed as lipid-associated macrophages (LAM) was discovered in both mouse and human adipose tissue characterized by TREM2 expression (65). Despite the fact that mice with TREM2 deficiency had fewer LAM macrophages in CLS, they exhibited accelerated obesity with massive adipocyte hypertrophy, insulin resistance, and hyperlipidemia upon HFD feeding (65). In addition, single-cell sequencing studies have shown that CD9^+^TREM2^+^ ATMs have more specific surface markers CD45^+^CD11b^+^CD11c^+^CD9^+^TREM2^+^ for better identification (70). In addition, a new subset of ATMs expressing Duffy antigen receptors for chemokines (DARC^+^ ATMs) was also discovered to be recruited to CLS in eWAT under obesity conditions (66). DARC^+^ATMs were generated in response to IL-22 stimulation and exhibited high levels of IL-22 receptor and M2-like anti-inflammatory properties to reduce adipose tissue inflammation in obesity (66).

**Table 1:** Newly identified adipose tissue subtypes.

Macrophages phenotype	Category	Location	Marker	Function	Reference
**MMe (metabolically activated macrophages)**	Recruited macrophages	CLS	ABCA1, CD36, PLIN2	Removing dead adipocytes through lysosomal exocytosis	(61) (62) (63)
**CD9^+^ macrophages**	Recruited macrophages	CLS	CD9, LPL, PLIN2, CD63, LAMP2, CD16, CD206	Promotion of obesity	(64)
**LAM (lipid-associated macrophages)**	Recruited macrophages	CLS	TREM2, LIPA, LPL, CTSB, CTSL, FABP4, FABP5, LGALS1, LGALS3, CD9, CD36	Preventing metabolic disorders when adipocyte homeostasis is lost	(65)
**DARC^+^ macrophages**	Recruited macrophages	CLS	DARC, Ly6C(low), M2-related marker(high)	Anti-inflammation and reducing immune cell infiltration.	(66)
**MFe^hi^ macrophages**	Resident macrophages	Intercellular space; CLS (a small number)	CD163, TFRC, HO-1, FTL1, FTH1, CP, SLC40A1, F4/80, CD11c(high), CD206(low)	Coping with iron metabolic disorders	(67) (68)

Other than CLS, several distinct ATM phenotypes in adipose tissue have been reported. For instance, in the intercellular space of adipose tissue, a distinct ATMs population known as “MFe^hi^” with higher cellular iron content and an iron-recycling gene expression profile was found (67). These “MFe^hi^” ATMs displayed M2-like alternatively activation markers such as CD163 and MGL1/2 and decreased M1 markers (67). As a result, MFe^hi^ ATMs can manage high iron loads by storing iron, regulating iron-handling genes, and protecting adipocytes from iron overload (68). More research is needed to characterized these newly discovered macrophage subtypes and to determine the potential mechanisms that link these cells to obesity and related metabolic disorders.

### 3.2 Role of ATMs in adipose tissue function

**Figure 2:**
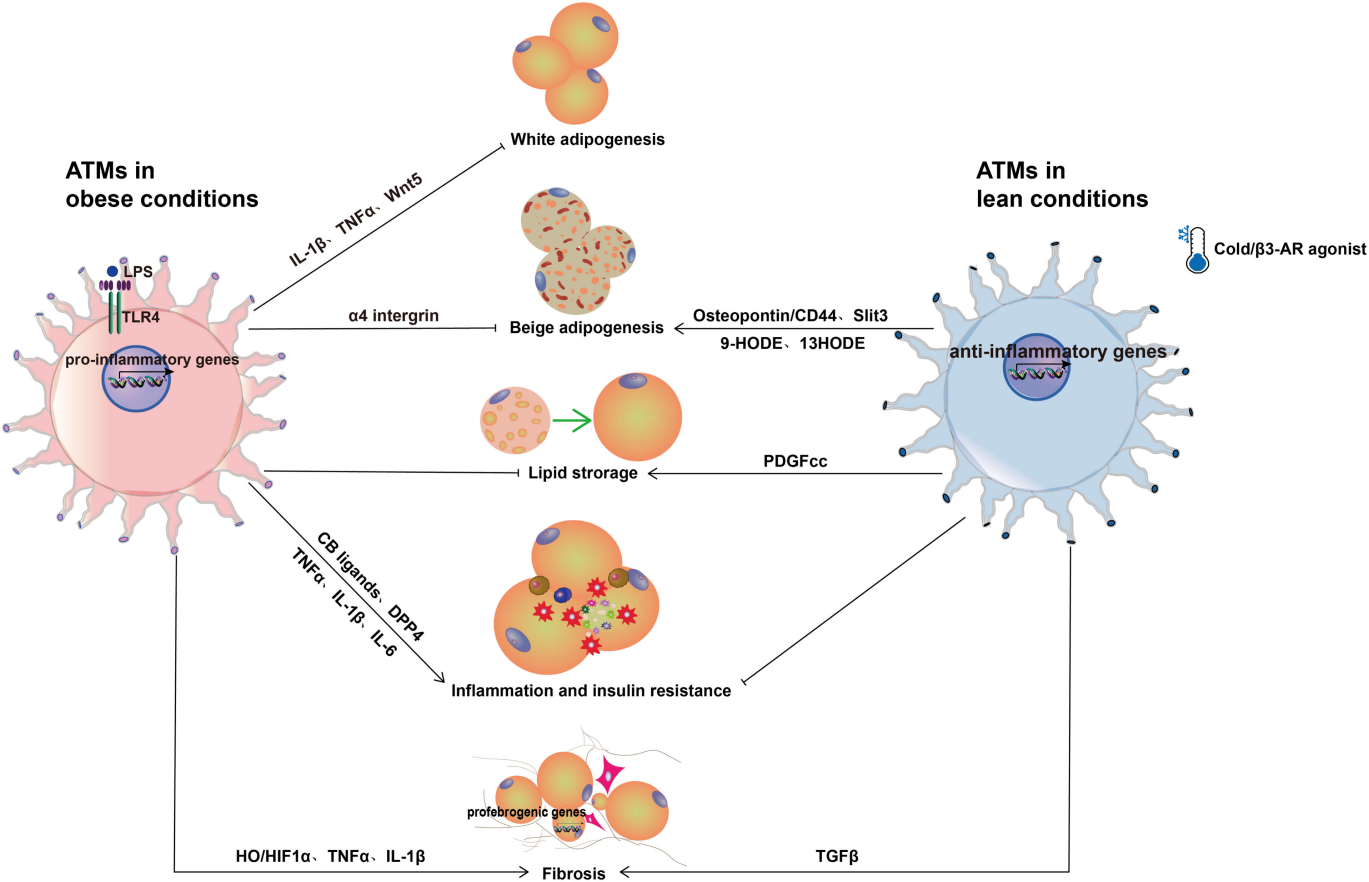
Role of ATMs in adipose tissue. In obese conditions, pro-inflammatory macrophages show detrimental effects on adipose tissue function such as inhibition of adipogenesis, promoting inflammation, insulin resistance, and fibrosis. The pro-inflammatory cytokines TNF-α and IL-1β and the protein factor Wnt5a inhibit preadipocyte differentiation when released by pro-inflammatory macrophages. In addition, TNF-α and IL-1β reduce the insulin sensitivity of adipocytes. Through LPS-TLR4 the LPS-induced CB ligands-CB1 signaling pathways, pro-inflammatory macrophages also aggravate adipose tissue inflammation. Moreover, macrophages secrete the enzyme DPP4, which causes both hyperglycemia and inflammation. In addition to inducing preadipocytes to produce abundant ECM, pro-inflammatory macrophages overproduce NO, which increases HIF-1α accumulation and promotes profibrogenic responses in preadipocytes, resulting in adipose tissue fibrosis. In lean conditions, ATMs are anti-inflammatory and play an important role in the formation and activation of beige adipocytes. In response to cold stimulation, ATMs polarize to an alternative activation state and promote the biogenesis of beige adipocytes *via* macrophage-secreted cytokine Slit3 and a sympathetic neuron-adipocyte signaling axis. Similar to cold stimulation, β3-AR agonists enhance the conversion of existing white adipocytes into beige adipocytes. Furthermore, β3-AR agonists induce alternative activation of macrophages to release osteopontin and the PPARγ ligands 9-HODE and 13-HODE, which stimulates beige adipocyte development.

The interactions between recruited pro-inflammatory macrophages and adipocytes are often harmful to the functions of adipocytes, including adipogenesis and lipid metabolism, inflammation, and related metabolic dysfunctions (**Figure 2**). In contrast, the resident macrophages in non-obese state are considered metabolically ‘favorable’ ATMs, which play important role in maintaining adipose tissue homeostasis *via* clearance of dead adipocytes. They are also critical for beige adipogenesis and thermogenesis, which lead to improved metabolic functions (**Figure 2**). Here we focus on reviewing the recent literature on ATMs and major adipose tissue functions.

#### 3.2.1 Role of ATMs in adipogenesis and lipid metabolism

The differentiation of preadipocytes to adipocytes is essential for the growth of adipose tissue in obesity. The expansion of white adipose tissue can dramatically enhance metabolic function and health. However, when immune cells, particularly pro-inflammatory macrophages, infiltrate adipose tissue, its potential to expand is inhibited. *In vitro* culture of preadipocytes with macrophage-conditioned medium elicits a pro-inflammatory response in both murine and human preadipocytes and impairs their differentiation to adipocytes (71–75), suggesting that macrophage-secreted factors contribute to its inhibitory effect on adipogenesis. Among the pro-inflammatory cytokines produced by macrophages, TNF-α and IL-1β have shown a direct inhibition on preadipocyte differentiation, however, neither TNF-α nor IL-1β neutralization reverses the anti-adipogenic effect of macrophage-conditioned medium (72, 76, 77), suggesting that other soluble factors could play a role. Wnt5a has been demonstrated to be expressed in human ATMs and circulating monocytes, and inhibition of Wnt5a activity in J774A.1 macrophage-conditioned medium improved mesenchymal precursor cells differentiation into adipocytes (78), suggesting that Wnt5a is a possible factor secreted by macrophages to suppress adipogenesis. Mechanistically, pro-inflammatory macrophages suppressed PPARγ activity in adipocytes by S-nitrosylation at cysteine 168, resulting in proteasome-dependent degradation of PPAR and decreased adipogenesis (79).

Beige adipose tissue is an inducible thermogenic type of adipose tissue that resides within white subcutaneous adipose tissue in mice and humans (80). Beige adipocytes can be induced by cold exposure, β3-adrenergic receptor (β3-AR) agonist, and PPAR ligands (81) *via* beige adipogenesis and white adipocytes conversion. Several studies have found that macrophages are critical players in the formation and activation of beige adipocytes (82–84). For instance, it has been demonstrated that pro-inflammatory macrophages directly interact with beige adipocytes *via* α4 integrin and VCAM-1, triggering a persistent inflammatory cycle in adipose tissue and inhibiting beige adipogenesis in obesity (85). In contrast, cold stimulation results in the production of the type 2 cytokines IL-4 and IL-13 by eosinophils, which activate macrophages and promotes the biogenesis of beige adipocytes (86). Furthermore, a recent study discovered the cytokine Slit3 secreted from anti-inflammatory macrophages promotes WAT beiging in response to cold *via* the sympathetic neuron-adipocyte signaling axis (87). In line with this discovery, subcutaneous WAT browning was significantly induced by injecting anti-inflammatory macrophages in obese mice induced by the HFD (88). However, a recent study found that conditionally and partially depleting adipose tissue CD206^+^ macrophages increased proliferation and differentiation of beige progenitors in normal and cold stimulated conditions (89, 90), suggesting that CD206^+^ ATMs inhibit beige adipogenesis. This might be as a result of mixed populations of CD206^+^CD11c^+^ and CD206^+^CD11c**^-^** ATMs present in CD206^+^ macrophages. More research is needed to determine which subtype has the inhibitory effect on beige adipogenesis. Similar to cold stimulation, β3-AR agonist is a potent inducer of the conversion of existing white adipocytes into beige adipocytes (91). Recent data also point to a role for resident macrophages in promoting beige differentiation in response to β3-AR activation through the clearance of dead adipocytes, the secretion of the chemokine osteopontin to recruit PDGFRα^+^CD44^+^ beige progenitors into subcutaneous adipose depot, and the production of the PPARγ ligands 9-HODE and 13-HODE *via* ALOX15 activity (92, 93). Overall, resident ATMs support beige adipogenesis and offer a potential therapeutic strategy to enhance metabolic health in obesity. More research is necessary to test these findings in human settings.

The classical function of adipose tissue is to store surplus energy as triglyceride during food intake and release free fatty acids during fasting. Several early *in vitro* studies reported that LPS-stimulated macrophages activate the lipolysis of 3T3-L1 adipocytes (94), which is accompanied by an inhibition of lipoprotein lipase (95) and a decrease in fatty acids synthesis (96). Moreover, LPS/IFNγ-activated macrophages are related to increased mitochondrial activity in human adipocytes, indicating that macrophage activation state may influence adipocyte bioenergetics (97). A recent study discovered that adipose tissue resident macrophages, rather than recruited CCR2^+^ macrophages, have an evolutionarily conserved role in lipid storage in adipocytes (98). In response to HFD feeding, these resident macrophages produce higher levels of PDGFcc, which promotes white adipocyte hypertrophy and hence prevents ectopic fat deposition in the liver and other tissues. Blocking PDGFcc reduces lipid accumulation in white adipocytes while increasing thermogenesis in brown adipocytes, indicating a vital role of PDGFcc in regulating lipid metabolism. Further study is needed to evaluate whether pharmacological inhibition of PDGFcc has therapeutic promise for obesity treatment.

#### 3.2.2 Role of ATMs in inflammation and related metabolic disorders

Increased ATM accumulation in obesity is one of the key contributors contributing to obesity-induced inflammation both locally and systemically. Newly recruited pro-inflammatory macrophages release a considerable amount of pro-inflammatory cytokines such as TNF-α, IL-6 and IL-1β and impede insulin signaling transduction in adipocytes (99–101). Consistently, the infiltration of pro-inflammatory macrophages precedes the IR in obese mice *in vivo* (6, 102), suggesting a causal role for inflammation in the development of IR in obesity. Insulin-resistant adipocytes release more free fatty acids and activate ATMs, resulting in a vicious loop that exacerbates inflammation *via* TLR4 (103). Moreover, TLR2 and TLR9 deficiency promotes HFD-induced adiposity, visceral adipose inflammatory responses, and IR in mice (104, 105), indicating that TLRs play a significant role in adipose tissue inflammation and IR in obesity. LPS derived from gut microbiota is another potential factor for inducing inflammatory responses in adipose tissue. On the one hand, LPS activates ATMs *via* TLR4 and amplifies inflammation by adipocyte-macrophage interactions (106). On the other hand, LPS causes robust productions of endogenous ligands for cannabinoid (CB) receptors in ATMs (107), which contributes to chronic inflammation in visceral adipose tissue, hyperglycemia, and IR (108). Furthermore, CB1 receptor blockage reduced LPS-induced pro-inflammatory responses in macrophages, alleviated adipose tissue inflammation and glucose intolerance (108, 109). In addition, other inflammatory mediators or proteins also contribute to adipose inflammation in obesity. DPP4, an enzyme that effectively increases blood glucose levels by degrading incretin peptides, was found to be more abundant in F4/80^+^ macrophages in CLS in adipose tissue than in adipocytes (110, 111). DPP4 inhibition dramatically reduced pro-inflammatory macrophage migration while producing an anti-inflammatory phenotype shift in adipose tissue macrophages, reducing obesity-induced inflammation and IR (112).

Additionally, pro-inflammatory macrophages play an important role in the development of adipose tissue fibrosis in obesity, which is another important pathogenic feature of obesity. Adipose tissue fibrosis is characterized by an increase in the expression and remodeling of extracellular matrix (ECM) proteins in WAT (113). The fibrotic deposition in adipose tissue has been observed as bundles of collagen fibers (Collagen I, III) in subcutaneous fat and thin fibrous lobule-like bands (Collagen VI) surrounding adipocytes in omental fat from subjects with obesity (114, 115). Collagens and fibronectin are expressed more abundantly in adipose tissue SV fractions than in adipocytes (114), indicating that SV fractions may be the primary cell types for fibrotic protein synthesis. Marcelin et al. have investigated the cellular origins of WAT fibrosis and discovered that pro-fibrotic cells originate from PDGFR^+^CD9^high^ cells within adipose tissue SV fractions (116). Human preadipocytes cultured *in vitro* with LPS-activated macrophages had a pro-inflammatory phenotype and produced abundant ECM consisting of collagen 1, tenascin-C, and fibronectin (77, 117). Furthermore, macrophages dramatically increased the levels of ECM breakdown enzymes such as matrix metalloproteinases in both preadipocytes and adipocytes *via* the pro-inflammatory cytokines TNF-α and IL-1β (118, 119). In contrast to *in vitro* studies, anti-inflammatory macrophages have been linked to increased adipose tissue fibrosis in individuals with IR (115). Mechanistically, TGF-β has been shown to induce myofibroblast-like cells from adipose tissue progenitor cells (preadipocytes) treated with ATMs (120). Hypoxia is an additional essential component contributing to adipose fibrosis. The expansion of adipose tissue in obesity is associated with adipose tissue hypoxia, as has been demonstrated in adipose tissue of several obese mouse models (*ob/ob*, KKAy, diet-induced) (121–123) and human subjects with obesity (124). Mechanistically, adipose tissue hypoxia increases HIF-1α expression and stability, which triggers profibrogenic transcription in preadipocytes (125). Furthermore, pro-inflammatory macrophages overproduced NO, which elevated HIF-1α accumulation and promoted profibrogenic responses in preadipocytes, resulting in adipose tissue fibrosis (126). Collectively, these findings suggest to the possibility of targeting pro-inflammatory macrophage-mediated inflammatory pathways to diminish obesity-induced inflammation, IR and fibrosis.

## 4 Targeting macrophages to improve metabolic health

**Figure 3:**
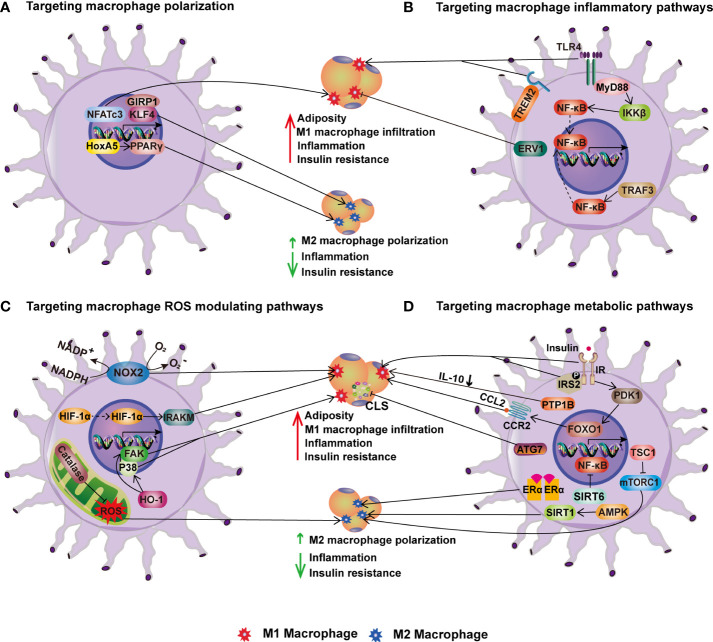
Targeting macrophages for improving metabolic health. **(A)** Targeting macrophage polarization. In HFD-induced obesity, transcription factors NFATc3, KLF4 and its coactivator GIRP1 enhance M1 macrophage polarization and infiltration into adipose tissue, inflammation, and insulin resistance. HoxA5 and PPAR, on the other hand, increase M2 macrophage polarization and thereby ameliorate obesity-induced inflammation and insulin resistance. **(B)** Targeting macrophage inflammatory pathways. TLR4-MyD88-IKK signaling and TRAF3 activation enhance adipose tissue M1 macrophage infiltration, inflammation, and IR in obesity *via* NF-B. On contrary, overexpression of ERV1 in macrophages reduces adiposity, hepatic and adipose inflammation, and hyperglycemia caused by HFD. **(C)** Targeting macrophage ROS modulating pathways. NOX2, HIF-1α, and HO-1 in macrophages increase obesity-induced adiposity, inflammation, and insulin resistance, whereas catalase inhibits inflammation *via* increasing M2 macrophage polarization. **(D)** Targeting macrophage metabolic pathways. The stimulation of macrophage insulin pathways such as IR-IRS2 and PDK1-FoxO1 signaling promotes HFD-induced obesity and insulin resistance. PTP1B, an insulin signaling negative regulator, induces IR by lowering IL-10. In contrast, mTORC1 activation improves M2 macrophage polarization and protects mice from HFD-induced obesity, inflammation, and insulin resistance. In addition, the ATG7-mediated autophagy pathway reduces CLS numbers and adipose tissue inflammation in obesity. Furthermore, other metabolic pathways regulated by ERα, SIRT1 and SIRT6 enhance M2 macrophage polarization, reducing inflammation and IR in obesity.

Given that ATMs play critical roles in both the onset and progression of obesity-related metabolic disorders, strategies that target the phenotypic flexibility of macrophages to fulfill tissue environment needs have demonstrated great therapeutic promise. The following is a summary of the prospective treatment targets for obesity and related metabolic diseases that can be delivered to macrophages (**Table 2**; **Figure 3**).

**Table 2:** Targeting macrophages for improving metabolic health.

Molecular targets	Approach	Phenotype
**IKKβ**	Myeloid cell specific IKKβ deletion (127)	↓ IR after HFD.
**TLR4**	Hematopoietic cell specific TLR4 deletion (128)	↓ IR, ↓adipose and liver inflammation
**Fas**	Myeloid/hematopoietic cell-specific Fas deletion (129)	↓ skeletal muscle IR, no effect on inflammation in liver and AT.
**MyD88**	Myeloid cell-specific MyD88 deletion (130)	↓ atherosclerosis, IR, and systemic inflammation after HFD.
**TRAF3**	Myeloid cell-specific TRAF3 deletion (131)	↓inflammation and IR in HFD-obese mice; ↑ inflammation in liver and adipose in lean mice.
**ERV1**	Myeloid cell-specific overexpression (132)	↓adiposity and inflammation after HFD.
**NOX2**	Myeloid cell-specific NOX2 deletion (133)	↓adiposity and adipose inflammation
**HIF1α**	Myeloid cell-specific HIF1α deletion (134)	↓systemic IR and inflammation after HFD
**HO-1**	HO-1+/- mice (135)	↓adiposity, adipose inflammation, and IR after HFD
**NFATc3**	NFATc3-/- mice (136)	↓hepatic steatosis and inflammation after HFD
**HoxA5**	HoxA5 overexpressed mice (137)	↓adiposity and inflammation after HFD
**Insulin receptor (IR)**	Mice with macrophage IR deletion (138)	↓IR after HFD
**PTPB1**	Macrophage PTPB1 deletion (139)	↓IR, liver damage and chronic inflammation
**IRS2**	Mice with macrophage IRS2 deletion (140)	↓adiposity and glucose intolerance after HFD
**mTORC1**	Myeloid cell-specific TSC1 deletion to constitutively activate mTROC1 (141)	↓obesity, glucose intolerance, and AT inflammation after HFD
**SIRT1**	Myeloid cell-specific SIRT1 deletion (142–145)	↓glucose tolerance, ↑ liver steatosis and AT inflammation
**TREM2**	TREM2 overexpressed mice (146)	↑AT inflammation, adiposity, IR after HFD.
**Catalase**	Global catalase deficiency (147)	↑oxidative stress, inflammation, and IR
**PPARγ**	Skeletal muscle and liver specific PPARγ depletion (148, 149)	↑IR in muscle and liver.
**KLF4**	Myeloid cell-specific KLF4 deletion (150)	↑adiposity, glucose intolerance, and IR after HFD
**GRIP1**	Myeloid cell-specific GRIP1 deletion (151)	↑ adipose inflammation, hyperglycemia, and IR
**ATG7**	Myeloid cell-specific ATG7 deletion (152)	↑adipose inflammation and hyperglycemia.
**PDK1/FoxO1**	Pdk1 deletion in macrophages; constitutive activation of nuclear Foxo1 (153)	↑ adipose inflammation and IR
**Estrogen receptor α (ERα)**	Macrophage ERα deletion (154)	↑adiposity, IR and atherosclerotic lesion area
**SIRT6**	Myeloid cell specific SIRT6 deletion (155)	↑ adipose and liver inflammation and IR
**PER1/PER2**	Myeloid cell-specific deletion of core clock genes Period1 (PER1) and Period2 (PER2) (156)	↑adipose inflammation and IR after HFD.

IR, insulin resistance; HFD, high-fat diet; ↑ increase; ↓ reduce.

### 4.1 Targeting macrophage polarization

ATMs have been shown to negatively modulate insulin action *via* CD11c^+^ pro-inflammatory macrophages (157), indicating that pro-inflammatory macrophages are a target for the treatment of obesity-related insulin resistance. Fatty acids are one of the major factors controlling the activation of ATMs.For example, saturated free fatty acids/TLR signalling, TNF/TNF receptor signalling induce the classically activation of macrophages (158–163), while unsaturated fatty acids like oleic acid, linoleic acid, DHA, and n-3 PUFA induce alternatively activated phenotype. Furthermore, omega-3 PUFA can increase lipolysis and fatty acid re-esterification in alternatively activated macrophages (164). These findings indicate that consuming unsaturated fatty acids may polarize ATMs to alternatively activated phenotype, thereby regulating lipid metabolism or alleviating the symptoms of obesity-related diseases.

Rosiglitazone, a PPARγ activator, also encourages alternatively activated macrophage infiltration into adipose tissue in mice receiving HFD (165–168). PPARγ deficiency in macrophages promotes the predominance of pro-inflammatory macrophages and the decrease of alternatively activated macrophages in adipose tissue in obesity (148, 169), indicating that PPARγ is essential in controlling macrophage alternative activation. Moreover, an intact IL-4 and IL-13 signaling is required for maturation of alternatively activated ATMs and reducing diet-induced obesity and IR in mice (170, 171). However, myeloid cell-specific knockout of IL4R alpha decreased insulin sensitivity in lean mice while improving parameters of glucose homeostasis and partially protecting against adipose tissue inflammation in obese mice (172), indicating IL-4R signaling likely plays a significant role in maintaining the alternative activation of macrophage in lean conditions but not in obesity.

A number of transcription factors have been found to influence ATMs polarization. For instance, Krüppel-like factor 4 (KLF4) has been demonstrated to promote monocyte differentiation *in vivo* (173). Moreover, KLF4 is strongly induced in alternatively activated macrophages by STAT6 while being reduced in pro-inflammatory macrophages by NF-kB inhibition (150). Consistently, KLF4-deficient macrophages displayed increased pro-inflammatory cytokine expression, and myeloid-specific KLF4 deficiency predisposed mice to diet-induced obesity, glucose intolerance, and IR (150), indicating a crucial role for KLF4 in regulating macrophage polarization and maintenance of adipose tissue homeostasis. Similar metabolic problems were brought on by the knockdown of the protein known as glucocorticoid receptor-interacting protein 1 (GRIP1), which acts as a coactivator for KLF4 (151). Contrarily, nuclear factors of activated T cells (NFATc3) play a different role in controlling the transcription of various genes in immune cells. Nfatc3-/- mice showed adipose tissue macrophage polarization toward alternative activation, which significantly reduced hepatic steatosis and inflammation in HFD mice, indicating the potential role of NFATc3 in promoting adipose tissue inflammation (136). Homeobox A5 (HoxA5), a developmental transcription factor, has been demonstrated to support adipocyte differentiation by inhibiting the PKA/HSL pathway (174). HoxA5 has also been shown to reduce endoplasmic reticulum stress and inflammatory responses in adipocytes by blocking the eIF2/PERK signaling pathway (137). Additionally, Hoxa5 transcriptionally activated the PPARγ pathway to promote alternative activation of macrophage and WAT browning (137), which in turn alleviated obesity-induced chronic inflammation. These findings imply that Hoxa5 may represent a promising therapeutic target for the management of obesity.

Notably, some therapeutic options and drugs have been developed to treat obesity-related metabolic diseases by regulating macrophage polarization. For obese patients who have failed to respond to exercise and dietary changes, bariatric surgery is an option. Studies have shown that after bariatric surgery, ATMs is biased toward the alternative activation with an increase of CD163 expression (40). However, subsequent research expressed concern on this notion, claiming that modifications in CD163-positive cells do not precisely reflect metabolic improvements following weight loss (175). Further research into the mechanism of bariatric surgery is required. Metformin, the most popular anti-diabetic medication, is crucial for macrophage polarization. Metformin was shown to decrease pro-inflammatory markers like CD11c and MCP-1 in the adipose tissue of HFD mice (176). Additionally, *in vitro* metformin treatment to pro-inflammatory macrophages improved metabolic disorders in brown adipocytes (177). Dipeptidyl peptidase-4 (DDP4) inhibitors Linagliptin and Sitagliptin are both used primarily to control blood glucose levels in patients with type 2 diabetes. These two drugs have been shown to decrease obesity-induced inflammation and IR by inhibiting pro-inflammatory and promoting alternative activated macrophages because DDP4 is largely expressed in pro-inflammatory macrophages and its expression was significantly increased in obese mice (112, 178). Similar mechanisms are shared by a number of sodium-glucose cotransporter 2 inhibitors, including empagliflozin. Through the phenotypic switch of macrophages to alternative activation in the liver and WAT, empagliflozin can reduce body weight by inducing WAT browning and reducing inflammation associated with obesity (179, 180). In conclusion, targeting macrophage polarization is a feasible and worthwhile direction that may benefit the vast majority of patients suffering from metabolic diseases.

### 4.2 Targeting macrophage inflammatory pathways

Adipose tissue inflammation is a major contributor to obesity-related metabolic diseases such as IR and hepatic steatosis. In adipose tissue, ATMs play dominant role in producing pro-inflammatory cytokines, which cause inflammation in obesity. NF-kB is one of the main masters of inflammatory responses. IKK is a crucial enzyme that activates NF-kB in myeloid cells. Mice with myeloid cell-specific IKKβ deletion preserved insulin sensitivity when fed with HFD (127). Furthermore, mice with hematopoietic cell-specific deletion of TLR4 demonstrated an improvement in peripheral insulin sensitivity after HFD feeding, which is associated with to a notable decrease in macrophage infiltration and inflammatory cytokines in both adipose tissue and the liver (128). MyD88, a TLR4 downstream signaling protein, is crucial in triggering inflammatory response. MyD88 deficiency in myeloid cells reduced macrophage infiltration to adipose tissue and their polarization to pro-inflammatory phenotype (130). Along with this, there is a considerable reduction in atherosclerosis, insulin resistance, and systemic inflammation induced by HFD feeding. Another typical intracellular signaling protein for TLRs is TNF receptor-associated factor 3 (TRAF3), which is anti-inflammatory in lean but pro-inflammatory in obese conditions. This is supported by research showing that myeloid cell-specific TRAF3 deletion reduced the number of macrophages in eWAT, as well as IR and the expression of pro-inflammatory cytokines in the liver and adipose tissue of obese mice (131). In contrast, TRAF3 deletion increased the expression of pro-inflammatory cytokines in the liver and adipose tissue of lean mice. Moreover, activation of the Fas signaling pathway may also be a crucial element of the inflammatory response. In HFD-induced obese mice, *ob/ob* mice, and mice acutely treated LPS, myeloid/hematopoietic cell-specific Fas-depletion preserved skeletal muscle insulin sensitivity, which was contributed by the decreased TNF-α levels in circulation (129). However, there was no difference in immune cell infiltration or local cytokine expression in adipose, liver, or skeletal muscle, indicating that the protective role of myeloid Fas depletion is more closely linked to a reduction of systemic inflammation.

Contrary to the inflammatory triggers listed above, it has been shown that TLR4 signaling from the triggering receptor expressed on myeloid cells 2 (TREM2) negatively modulates the inflammatory response in macrophages (181). A recent study has found that TREM2 may be involved in the inflammatory response in adipose tissues. Following HFD feeding, mice with TREM2 overexpression showed elevated macrophage and T cell recruitment into adipose tissue as well as increased adiposity, IR, and hepatic steatosis (146). These findings suggest that TREM2 acts as a novel regulator of adipogenesis and that inhibiting TREM2 signaling may be a therapeutic target for obesity and IR. To fully understand the underlying mechanisms of TREM2 in regulating the inflammatory response in adipose tissues, additional research on macrophage-specific deletion of TRME2 is required. Moreover, endogenous lipids known as specialized pro-resolving mediators (SPMs), which include resolvins, protectins, and maresins, mediate the resolution of inflammation (182). Mice overexpressing the human resolvin E1 receptor (ERV1) in myeloid cells displayed reduced adiposity, hepatic and adipose inflammation, and hyperglycemia induced by HFD (132). Resolvin E1, a natural ERV1 agonist, administration replicated the pro-resolving effects obtained from ERV1 overexpression. This protective metabolic impact is in part explained by systemic activation of resolution programs leading to increased synthesis of specialized pro-resolving mediators. Taking together, targeting inflammatory pathways in macrophages offers a great potential for controlling adipose tissue inflammation and the ensuing metabolic disorders induced by obesity.

### 4.3 Targeting reactive oxygen species modulating pathways in macrophages

Oxidative stress and chronic inflammation are the important underlying factors for obesity-associated metabolic diseases. The imbalance between the oxidative and anti-oxidant systems of the cells and tissues results in the overproduction of oxygen free radicals and reactive oxygen species (ROS). Oxidative stress increases lipid peroxidation products, protein carbonylation which leads to cellular dysfunction. As the NADPH oxidase catalytic subunit, NOX2 has been demonstrated to be involved in obesity-induced IR, hyperlipidemia, and liver steatosis (183). Mice lacking myeloid-NOX2 showed reduced adiposity, adipose inflammation, and macrophage infiltration compared to controls when given a 16-week HFD diet (133). These results support the idea that NOX2 signaling in macrophages plays a role in the pathogenesis of obesity-induced metabolic disorders. Potentially, obesity may be reduced by targeted suppression of monocyte/macrophage NADPH oxidase in adipose tissue to maintain metabolic function.

Hypoxia is also a factor in the increased oxidative stress associated with obesity. The transcription factor hypoxia inducible factor-1 (HIF-1) regulates the expression of numerous hypoxic responsive genes by nuclear translocation and mediates adaptive responses to oxidative stress. HIF-1α has been demonstrated to contribute to oxidative stress and fibrosis in obese people (184). Additionally, macrophages in CLS and adipocytes are both hypoxic and inflammatory (185). In fact, mice with myeloid-specific HIF-1α deletion had enhanced adipose tissue vasculature development, which mitigated systemic IR and HFD-induced inflammation (134). Furthermore, a recent study identified interleukin-1 receptor-associated kinase M as the mechanism underlying HIF-1α-induced adipose tissue dysfunction in obesity (186), supporting the notion that HIF-1α in myeloid cells is crucial to obesity-related pathological growth of adipose tissue and systemic IR.

Additionally, heme oxygenase-1 (HO-1) is a stress-inducible enzyme that is crucial in several pathophysiological conditions, particularly inflammation and oxidative damage. Heme oxygenase (HO-1) expression was highly induced in the visceral adipose tissue, especially the SV fraction of HFD-fed mice. Myeloid HO-1 haploinsufficiency attenuated HFD-induced adiposity, adipose inflammation, and IR, due to impaired macrophage migration toward adipose tissue and reduced angiogenesis (135). Mechanistically, HO-1+/- macrophages displayed decreased chemoattractant-induced p38 phosphorylation and focal adhesion kinase expression (135). These findings point to a unique role of the myeloid cell HO-1 in adipose macrophage infiltration and IR development during obesity.

In contrast to the preceding factors, catalase, an important oxidative stress regulator, has been shown to control ATM polarization under both resting and metabolic stress conditions. Global catalase deficiency or use of the catalase inhibitor 3-aminotriazole causes oxidative stress, increased inflammation and IR in both lean and HFD-induced obese mice (147). Catalase inhibition increased pro-inflammatory macrophage accumulation but decreased alternatively activated macrophage accumulation in eWAT, indicating that endogenous catalase may be a critical regulator of obesity-related inflammation and IR.

### 4.4 Targeting macrophage metabolic pathways

Obesity-associated metabolic problems appear to be caused by a combination of metabolic endotoxemia and metabolic stress induced by chronic exposure to excessive amounts of nutrients. Because immune cell metabolism and function are inextricably connected, addressing the different metabolic pathways of macrophages could provide a unique opportunity to modify its phenotype and subsequent biological roles in obesity.

#### 4.4.1 Insulin pathway as a target

Despite previous research, the main impact of macrophage insulin action on obesity and related metabolic disorders is still debated. Mice lacking macrophage insulin receptor were protected from the onset of obesity-related IR after HFD feeding (138). This protection was accompanied by lower macrophage counts in WAT and serum tumor TNF-α levels, which reflect a marked decrease in the local and systemic inflammation linked to obesity. These findings suggest that insulin action in myeloid cells plays an unexpectedly important role in regulating macrophage invasion into WAT and the development of obesity-associated IR. In line with this study, mice with macrophage insulin receptor substrate 2 (IRS2) deletion demonstrated protection from HFD-induced obesity and glucose intolerance due to increased energy expenditure *via* enhanced BAT activity and WAT beiging (140). Additionally, IRS2-deficient macrophages exhibited a transcriptional profile that was anti-inflammatory (140), indicating a crucial role for macrophage IRS2 signaling in ATM polarization and energy homeostasis. These findings may open therapeutic opportunities for the treatment of obesity. However, protein tyrosine phosphatase-1B (PTP1B), an intracellular protein that inhibits insulin and leptin signaling, has been shown to promote inflammation caused by obesity. Mice deficient in macrophage PTP1B displayed improved glucose and insulin tolerance, reduced liver damage and chronic inflammation after HFD feeding (139). The beneficial effect of PTP1B deletion in macrophages is due to increased IL-10 levels, which are inversely related to serum insulin and alanine transferase levels. These findings suggest that inhibiting myeloid PTP1B could be used to treat obesity-related inflammation and diabetes.

#### 4.4.2 Nutrient sensing pathways as a target

Many studies have been conducted on the function of mTORC1 in obesity and associated inflammation. These studies have demonstrated the link between mTORC1 activation and obesity. Despite having no impact on the HFD-induced obesity, pharmacological mTORC1 inhibition by rapamycin worsened the inflammation and glucose intolerance, as shown by the rise in adipose tissue pro-inflammatory macrophages and elevated mRNA levels of pro-inflammatory cytokines such as TNF-α, IL-6, and MCP-1 (187). Additionally, macrophages derived from bone marrow exhibited pro-inflammatory phenotype as a result of *in vitro* mTORC1 inhibition (187). These results suggest that mTORC1 activity is a key regulator of macrophage plasticity and inflammation in adipose tissue. To further investigate the role of myeloid cell mTORC1 activation in obesity-induced inflammation, mice with myeloid cell specific TSC1 deletion and thus constitutive mTORC1 activation were generated. Mice lacking Tsc1 in macrophages exhibited protection from HFD-induced obesity, glucose intolerance, and adipose tissue inflammation (141). This protection was accompanied by mTORC1-dependent alternative activation of macrophages, indicating a protective role for mTORC1 activation in HFD-induced obesity and metabolic disorders. Unlike mTORC1, myeloid cell deficiency of mTORC2 obtained by Rictor deletion had no impact on HFD-induced obesity, adipose tissue inflammation, or systemic IR (188). However, mice lacking Rictor showed increased susceptibility to LPS-induced septic shock, indicating that mTORC2 is more important in diseases associated with severe inflammation than obesity-induced chronic low-grade inflammation.

Autophagy, a crucial cellular response pathway for sensing nutrient levels, is essential for cell survival and metabolism. When bred to *ob/+* mice to induce metabolic stress, mice with myeloid cell-specific deletion of autophagy-related 7 (ATG7) displayed increased CLS numbers, activated NLRP3 inflammasome and IL-1β production in adipose tissue, as well as hyperglycemia (152). This was attributed to mitochondrial dysfunction in autophagy-deficient Macrophages, suggesting a critical role for macrophage autophagy in regulating adipose inflammation and insulin sensitivity in obesity.

As one of the key pathways regulating glucose and energy homeostasis, the 3-phosphoinositide-dependent protein kinase 1 (PDK1)/forkhead transcription factor (FoxO1) pathway has also been investigated in adipose tissue macrophages. PDK1 deletion in macrophages resulted in increased pro-inflammatory macrophages in adipose tissue and IR, which was reversed by inactivating nuclear FoxO1 (153). Furthermore, constitutively activating nuclear FoxO1 increased pro-inflammatory macrophages in adipose tissue *via* CCR2 and IR on HFD (153). Accordingly, PDK1 inhibits FoxO1 to regulate macrophage infiltration, and the PDK1/FoxO1 pathway in macrophages is essential for regulating macrophage polarization and insulin sensitivity in obesity.

Additionally, estrogen receptor alpha (ERα) plays a significant role in the control of glucose homeostasis (189). Even with a normal diet, mice with myeloid-specific ERα deletion displayed increased adiposity, IR, and atherosclerotic lesion area (154). Moreover, ERα deficiency reduced the response of isolated macrophages to IL-4-mediated alternative activation but promoted the inflammatory response to palmitate (154). This suggests that macrophage ER is important for suppressing inflammation and maintaining insulin sensitivity, making it a potential therapeutic target to combat obesity and IR.

#### 4.4.3 Sirtuins as a target

Myeloid cell Sirtuin 1 (SIRT1) has been shown to play a protective role in studies of metabolic diseases caused by obesity. When given an HFD, mice with myeloid cell *Sirt1* deletion exhibited pro-inflammatory macrophage polarization in adipose tissue and increased adipose tissue macrophage hypoxia and inflammatory response (142–144), which impaired glucose tolerance and exacerbated liver steatosis (143, 145). In line with this, dietary quercetin has been demonstrated to reduce macrophage infiltration, control macrophage polarization, and regulate inflammation through the AMPK1/SIRT1 pathway, resulting in a reduction in HFD-induced IR and an increase in glucose uptake in adipose tissue (190). Similar to SIRT1, myeloid cell-specific SIRT6 knockout mice displayed increased pro-inflammatory macrophage infiltration in adipose and liver, as well as decreased insulin sensitivity *via* the NF-κB/STAT3 signaling pathway (155). These findings indicate that SIRT1 or SIRT6 in macrophages may be potential targets for combating obesity-induced tissue inflammation and IR.

#### 4.4.4 Circadian pathways as a target

Numerous studies have linked metabolic disorders like obesity to circadian clocks. Circadian clock dysregulation induces pro-inflammatory macrophages and potentiates adipose tissue inflammation in mice with Period1 (PER1) and Period2 (PER2) deletion in macrophages, according to a previous study (156). High MCP-1 levels in mice with myeloid cell-specific PER1/PER2 disruption attracted pro-inflammatory macrophage infiltration and increased inflammation and IR in HFD-induced adipose tissue (156). Mechanistically, PPARγ2 levels were decreased in PER1/2-disrupted macrophages and restoration of PPARγ2 levels reduced the infiltration of pro-inflammatory macrophages in adipose tissue, suggesting that PPARγ may link the molecular clock genes and obesity-related inflammation.

## 5 Concluding remarks and perspectives

Increased ATMs are the major contributor to adipose tissue inflammation in obesity. Efforts have been made to target macrophage recruitment to improve metabolic health and have shown a great promise in obese mouse models. For instance, blocking CCL2-CCR2 has been shown to reduce macrophage recruitment in adipose tissue and mitigated the obesity-induced inflammation and IR. Moreover, a dual CCR2/CCR5 antagonist reduced macrophage-mediated inflammation and prevented IR, providing a therapeutic potential for metabolic diseases linked to obesity. Another promising strategy is to promote the polarization of ATMs toward alternative activation. Several transcription factors, including PPARγ, KLF4, and HoxA5, have been shown to promote alternative activation of macrophages in adipose tissue and could be potential pharmacological targets. Additionally, strategies at targeting myeloid TLR4/NF-κB-mediated inflammatory pathways, ROS generating enzyme NOX2 and hypoxia adaptation factor HIF1α, and factors regulating glucose metabolism also appear to have a positive impact (**Table 2**; **Figure 3**). Further research is needed to validate the findings of mouse studies in humans.

The recent single cell RNA-sequencing studies have identified a broad spectrum of ATM subtypes, suggesting a heterogeneity and functional plasticity of ATMs in obesity. It remains to be determined the differences in the development, phenotype, and function of these newly discovered macrophages within adipose tissue. Also, understanding the regulatory factors and intracellular pathways that underpin functional differences between subtypes would provide new molecular targets. Finally, the development of new technologies that can target specific macrophage subtypes would considerably boost the translational potential of the aforementioned findings for the treatment of obesity and metabolic diseases.

## Author contributions

Conceptualization: DG, HG and SL; literature search: XL, YR, KC, WW, and DG; writing: XL, YR, and DG; review and editing: XL, YR, KC, WW, DG, HG, and SL. All authors contributed to the article and approved the submitted version.
